# Nitrous Oxide and Chloroform as an Anesthetic

**Published:** 1883-10

**Authors:** Alex. Boggs


### Nitrous Oxide and Chloroform as an Anesthetic.

If laughing gas has the advantage of producing anesthesia rapidly, it has the great drawback, when its use is prolonged, to produce asphyxia. In mixing eighty-five parts of nitrous oxide with fifteen parts of oxygen a perfectly safe mixture is obtained, but producing anesthesia only in proportion as the gas inhaled is under a pressure superior to that of the atmospheric air, as has been demonstrated on more than one occasion by Prof. Paul Bert. Dr. De Saint-Martin has succeeded in rendering this mixture effective at the ordinary pressure by adding six to seven grams of chloroform to each hectoliter. In trying the effects of this mixture on himself, Dr. De Saint-Martin had been able to convince himself that the anesthesia thus produced is very rapid, free from the power of exciting, and is unaccompanied by the irritating action which pure chloroform produces on the respiratory organs. —*Alex. Boggs, in Louisville Med. News.*

